# Nine Novel Phages from a Plateau Lake in Southwest China: Insights into *Aeromonas* Phage Diversity

**DOI:** 10.3390/v11070615

**Published:** 2019-07-05

**Authors:** Meng Bai, Ya-Hui Cheng, Xue-Qin Sun, Zi-Yi Wang, Yong-Xia Wang, Xiao-Long Cui, Wei Xiao

**Affiliations:** 1 Yunnan Institute of Microbiology, School of Life Science, Yunnan University, Kunming 650091, China; 2 Yunnan Engineering Laboratory of Soil Fertility and Pollution Remediation, Yunnan Agricultural University, Kunming 650201, China; 3 State Key Laboratory for Conservation and Utilization of Bio-Resources in Yunnan, Yunnan University, Kunming 650091, China; 4 Key Laboratory of the University in Yunnan Province for International Cooperation in Intercellular Communications and Regulations, Yunnan University, Kunming 650500, China

**Keywords:** bacteriophage, *Aeromonas*, genome evolution, large terminase, auxiliary metabolism gene

## Abstract

*Aeromonas* species are common pathogens of fish and some of them can opportunistically cause infectious diseases in humans. The overuse of antibiotics has led to the emergence of bacterial drug-resistance. To date, only 51 complete genome sequences of *Aeromonas* phages are available in GenBank. Here, we report the isolation of nine *Aeromonas* phages from a plateau lake in China. The protein cluster, dot plot and ANI analyses were performed on all 60 currently sequenced *Aeromonas* phage genomes and classified into nine clusters and thirteen singletons. Among the nine isolated phages, the DNA-packaging strategy of cluster 2L372D (including 2L372D, 2L372X, 4L372D, 4L372XY) is unknown, while the other five phages use the headful (P22/Sf6) DNA-packaging strategy. Notably, the isolated phages with larger genomes conservatively encode auxiliary metabolism genes, DNA replication and metabolism genes, while in smaller phage genomes, recombination-related genes were conserved. Finally, we propose a new classification scheme for *Aeromonas* phages.

## 1. Introduction

*Aeromonas* species are Gram-negative bacteria, widely distributed in various environments, including water, food and soil. They are common pathogens of fish and some of them are linked with human infectious diseases, for example, *Aeromonas hydrophila* could cause serious human infections such as bacteremia, pneumonia, endocarditis, empyema, arthritis, peritonitis, skin and soft-tissue infections [1,2,3] and fish diseases including red sore disease and ulcerative infections, which are responsible for huge economic losses in the aquaculture industry [4,5]. The multiple-antibiotic-resistant *A.hydrophila* has been reported in Korea and the USA, some of them contributing to huge economic losses in the aquaculture industry [6,7]. However, it has been reported that the third- and fourth-generation cephalosporins and fluoroquinolones were effective against most of the infections caused by *Aeromonas* species. The increasing antibiotic resistance issue also raised the concern in treatment of *Aeromonas* infections [7,8,9,10]. Phage therapy has been considered as a complementary or alternative therapy to treat bacterial infections besides antibiotics [11]. However, bacteriophages are considered to specifically infect their host bacteria species, therefore, phage cocktails are usually used in therapeutic treatments to extend the hostspectrum and overcome phage-resistant bacterial hosts [12,13].

On 1 April 2019, 51 complete genome sequences of *Aeromonas* phages were available in GenBank (to the best of our knowledge, listed in Table 1), 19 of which infect *Aeromonas hydrophila*, 25 infect *Aeromonas salmonicida*, and seven infect other *Aeromonas* species. Taxonomically, the majority of *Aeromonas* phages are double-strand DNA phages belonging to the *Myoviridae*(33/51), *Podoviridae* (7/51) and *Siphoviridae* (5/51) families, along with one ssDNA virus and five unclassified bacteriophage [5,14,15,16,17,18]. According to the classification of the International Committee on Taxonomy of Viruses (ICTV), *Aeromonas* phages in the *Myoviridae* family are further classified into the *Secunda5virus* genus (Aes012, Aes508, phiAS4, phage 31, and phage 25), the *Tevenvirinae* subfamily(Aeh1, Ah1 and AS-zj), and the *Biquartavirus* genus (phage 44RR2.8t), along with some unclassified *Myoviruses*. Phages in the *Siphoviridae* family are classified as either *T5virus* genus (AhSzw-1, AhSzq-1), *Pis4avirus* genus (pIS4-A), or *Hk97virus* genus (AsXd-1). Phages in the *Podoviridae* family are further classified as an *Autographivirinae* subfamily (AS7, 25AhydR2PP and CF7) or as unclassified *Podoviruses* (Ah1, vB_AsoP_Ca, ZPAH7B, ZPAH7). There are 34 *Aeromonas* phages that have yet to be assigned a genus or subfamily. *Aeromonas* phages appear to be remarkably heterogenous.

In the present study, we aim to expand our knowledge of existing *Aeromonas* phage diversity by isolating and characterizing the genome of nine phages from a plaque lake in China and give an overview of genomic prospect of the 60 sequenced *Aeromonas* phages. The comparative genomics, protein cluster, dot plot and ANI analyses were performed to group phages into clusters. In some of the clusters, phages infect different host species grouped together and showed high similarities with each other. This information could potentially provide reference on phage selection against *Aeromonas* species and prepare relative broad-spectrum phage cocktails by combining phages from different clusters. Additionally, we propose a new classification scheme for *Aeromonas* phages.

## 2. Materials and Methods

### 2.1. Phage Isolation

*Aeromonas hydrophila subsp. hydrophila* L372, *Aeromonas hydrophila subsp. hydrophila* 4572, *Aeromonas rivipollensis* D05, and *Aeromonas rivipollensis* 4512 were isolated by our lab and used as hosts. Sediment samples and water samples were collected from the Dianchi Lake (Yunnan province, China) on 1 June 2016. Sample was mixed with 5 g sediment sample, 50 mL water sample, 100 mL TSB and 0.2% (*v*/*v*) host mixture (equal volume of *A. hydrophila* strains L372 and 4572 and *A. rivipollensis* stains 4512 and D05 with 10^9^ CFU/mL), then incubated at 28 °C overnight and agitated at 160 r/min. Following incubation, the cultures were centrifuged for 10 min at 3200× *g* and the supernatants were filtered through 0.22 µm filters (Millipore, Burlington, MA, USA). Phages were isolated and purified using standard plaque assays after resuspension in TSB using *A. hydrophila* L372, *A. hydrophila* 4572, *A. rivipollensis* D05 and *A. rivipollensis* 4512 as hosts. The purified phages were stored in TSB and 30% glycerol (*v*/*v*) at −80 °C for future use.

### 2.2. Morphology

Transmission electron microscopy was used to observe the phages. Briefly, phage suspension (1.0 × 10^11^ PFU/mL) was placed on individual copper grids, and then negatively stained with 2% sodium phosphotungstate and examined by transmission electron microscopy (JEM-2100) at the Advanced Analysis and Measurement Center of Yunnan University.

### 2.3. Phage DNA Extraction, Sequencing and Annotation

The propagated and purified phage stocks (10^9^ PFU/mL) were centrifuged and filtered through 0.22 µm filter, then treated with 10 U/µL DNase I and RNase A (TakaRa, Dalian, China) and incubated at 37 °C for 1 h. The genomic DNA of the propagated phages was extracted using the TIANamp Virus DNA/RNA Kit (TIANGEN, Beijing, China) following the manufacturer’s instructions. The genomic DNA of phages 2L372D, 2L372X, 4L372X, 4L372XY, 44512, 44572, and 4D05 was sequenced at the Majorbio Cooperation (Shanghai, China) using Illumina HiSeq 2000. The resultant reads were assembled using SOAP denovo version 2.04 into a single contig for each phage [19]. The genomic DNA of phages 2D05 and 4L372D was sequenced using PacBio RS II. The resultant reads were assembled using SMRT Analysis version 2.3.0.

The genomes of nine phages were scanned for potential open reading frames (ORFs) with GeneMarkS (http://topaz.gatech.edu/GeneMark/) [20]. Annotation was carried out by comparing translated ORFs in BLASTP (http://blast.ncbi.nlm.nih.gov/Blast.cgi) [21,22]. tRNA genes were predicted by tRNAsan-SE version 1.21 [23].

### 2.4. Comparative Genomics

Genome comparisons were performed using EasyFig 2.2.2 [24] based on the results of tBLASTx [21] sequence comparisons. The minimum identity cutoff setting was 33%.

### 2.5. Phylogenetic Tree of terL

To predict the packaging strategy of analyzed phages, the method proposed by Casjens and Gilcrease was adopted [25]. The *terL* (terminase large subunit) protein sequences of 80 reference phages with experimentally identified packaging strategies were selected based on previously published studies [18,25]. Twenty-eight sequences of other *Aeromonas* phages (with annotated *terL*) were also included to give an overview of the evolution strategy of *Aeromonas* phages *terL*. Protein sequences of all *terL* were retrieved from the National Center for Biotechnology Information (NCBI) database, and accession numbers were provided in Supplementary Materials Table S4. Multiple sequence alignments of these terminase sequences were performed by PROMALS3D [26]. The IQ-TREE web service was used to generate an ML (Maximum-Likelihood) tree using the substitution model VT+F+R6 (evaluated and selected by IQ-TREE) and 1000 ultrafast bootstraps [27]. The tree was midpoint rooted by FigTree version 4.14.6 (http://tree.bio.ed.ac.uk/software/figtree/).

### 2.6. Protein Analysis

Protein cluster analysis was performed for nine isolated phages and 60 *Aeromonas* phages (9 isolated together with 51 GenBank available, accession numbers were provided in Table 2 and Table S3). Protein clusters were generated by GET_HOMOLOGUES version 3 [28] in default setting (minimum 75% coverage in BLAST pairwise alignments [22], minimum 1% sequence identity and maximum E-value of 1 × 10^−5^). Protein clusters statistics were created in Microsoft Excel and then visualized by heatmap package in R.

### 2.7. Dot Plot Analysis

Dot plot comparisons of phage genomes were performed using matrix dot plot generating computer program Gepard [29] at word length 10 and default settings of the other parameters.

### 2.8. ANI Analysis

The Average Nucleotide Identity (ANI) analysis was performed using online service JSpecies (http://jspecies.ribohost.com/jspeciesws).

## 3. Results

### 3.1. Morphology

**Figure 1:**
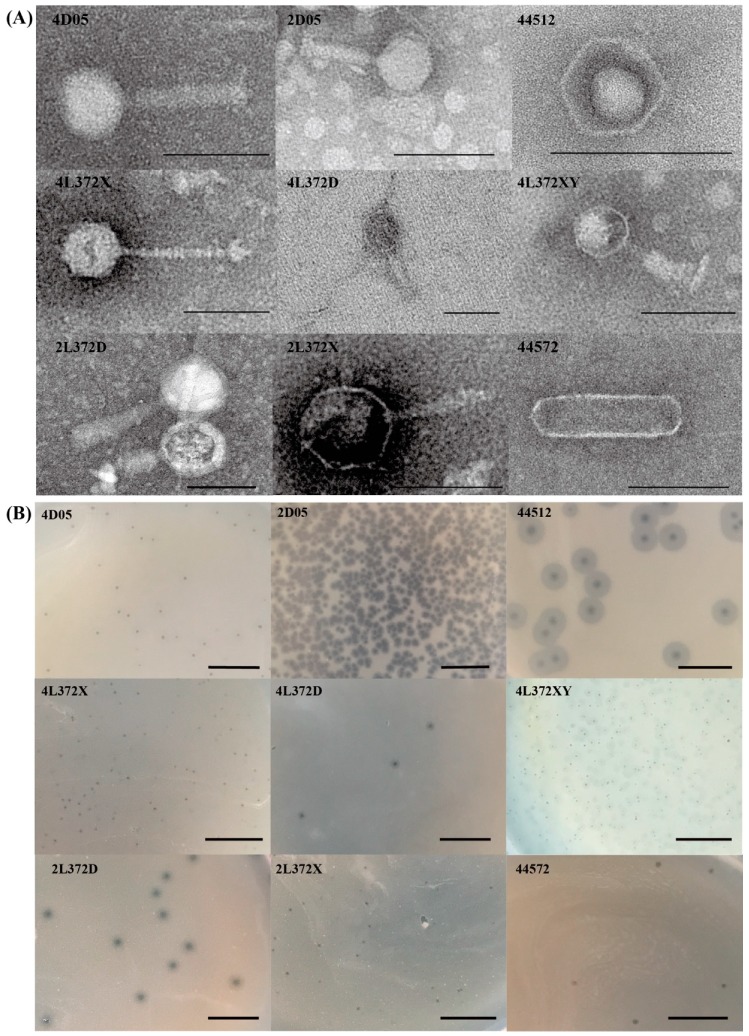
Morphologies of nine phages isolated in this study. (**A**) TEM micrographs: the phages were negatively stained with 2% sodium phosphotungstate, and the bars represent 100 nm in length. (**B**) Plaques of nine phages isolated in this study. The bars represent 1 cm in length.

Nine phages were isolated and purified using four *Aeromonas* bacteria strains (*A. hydrophilasubsp. hydrophila* strains L372 and 4572; *A.revipollensis* strains D05 and 4512) which were isolated and characterized previously by our lab. The nine phages were named as 2L372X, 2L372D, 4L372D, 4L372X, 4L372XY, 44572, 2D05, 4D05 and 44512. Phages 2L372X, 2L372D, 4L372D, 4L372X, 4L372XY were isolated using *A. hydrophila subsp. hydrophila* strains L372, phage 44572 was isolated using *A. hydrophila subsp. hydrophila* strains 4572, phages 2D05 and 4D05 were isolated using *A.revipollensis* D05, and phage 44512 was isolated using *A.revipollensis* 4512. From the TEM microscope (Figure 1A), the capsid diameter of the nine phages ranged from 43 nm to 81 nm. Six phages (2L372X, 2L372D, 4L372D, 4L372XY, 2D05, 4D05) belonged to the *Myoviridae* family, each of them with a polyhedron capsid and a contractile tail (tail length 65–114 nm). One phage (4L372X) showed a non-contractile tail with 102 nm in length, which was similar to a member of *Siphoviridae* family. Another phage (phage 44512) showed no tail in the TEM photo and no tail-related protein coding gene was annotated. Another phage (phage 44572) possessed an unusual elongated head of 165 nm in length and 51 nm in width, and no noticeable tail was observed in the TEM photo—although a putative tail fiber-related protein gene was annotated in its genome. The TEM morphology features, host strain, plaque, and sequencing and assembly information of each phage are summarized in Table 1. A photo of the plaque morphologies of nine phages is shown in Figure 1B.

### 3.2. Genomic Characterization

The characterization and sequencing statistics of nine phage genomes are shown in Table 2. The genome sizes ranged from 41,963 to 138,690 bp, encoded 63–252 ORFs, and had GC% varying from 35.1 to 56.4%. Three *A. rivipollensis* phages possessed a small genome of about 40 kb. Most of the *A. hydrophila* phages had a larger genome above 100 kb, except phage 4L372X which had a genome size of 45,698 bp. Another remarkable feature was the pronounced divergence in GC% between the nine phages. In Table 2, the first four *A. hydrophila* phages 2L372X, 2L372D, 4L372D, 4L372XY had a lower GC% of 35.54±0.43%, and phages 4L372X and 44572 had a medium GC% of 48.7% and 42.5% respectively. By contrast, the genomes of *A. rivipollensis* phages 2D05, 4D05 and 44512 obtained a relatively higher GC% of above 52%. The GC% of all nine phages was lower than the host GC%.

### 3.3. tRNAs

Of the nine isolated *Aeromonas* phages, the genome of phage 44572 encoded 20 tRNA-like genes, 12 of which were canonical tRNA that potentially carry amino acids, and eight of which were pseudo tRNA genes. However, the other eight phages encoded fewer or no tRNA genes compared to phage 44572 (Table 2). Previous studies showed that the phage genome encodes tRNA genes that permit the phages to presumably bypass codons overused in the phage genes, since the repertoire of tRNAs from the host is likely inadequate to translate phage mRNAs efficiently [30,31]. In our study, the tRNAs genes, together with pseudo tRNA genes in 44572, corresponded to 16 of the total 20 amino acid types. There was a possibility that phage 44572 would tend to partially use its own translation machinery instead of fully relying on the host translation machinery.

### 3.4. Comparative Genome Analysis

The genome homology of the nine phages varied based on nucleotide identity. The pairwise nucleotide identity of nine phages, according to BLASTn [22] analysis, is shown in Table 3. In the results, *A. hydrophila* phages 4L372D, 2L372D, 2L372X and 4L372XY showed relatively high pairwise nucleotide identity, while phages 44572 and 4L372X did not exhibit obvious similarities with the other phages. *A. rivipollensis* phages 2D05 and 4D05 showed high nucleotide identity.

**Figure 2:**
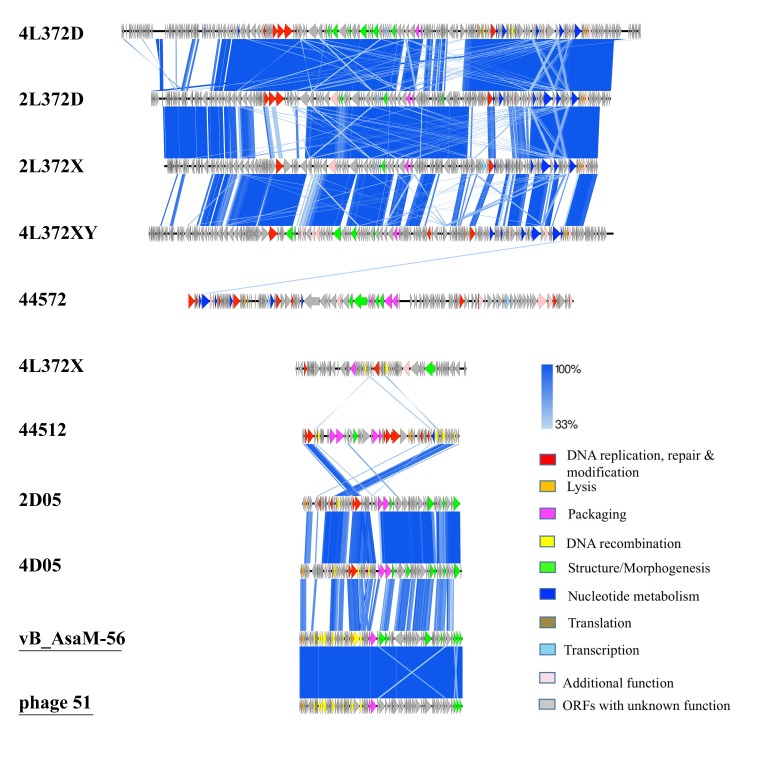
tBlastX comparison of the nine phage genomes. Arrowheads denote genes, and the identify cut-off is set as 33%. Predicted open reading frames (ORFs) are marked with arrows and functions are indicated in different colors: DNA replication and modification (red); DNA recombination (yellow); Transcription (light blue); lysis/lysogeny (dark yellow); structure/morphogenesis (green); packaging (pink); Nucleotide metabolism (dark blue); additional function (light pink). ORFs with unknown function are in light grey. The reference phages most related to the novel phages were included and underlined.

To visualize regions sharing similarities across the nine novel phage genomes, pairwise comparison of the nine phage sequences was performed by tBLASTx [21] (Figure 2). The resulting map was consistent with BLASTn analysis, as shown in Table 3. Regions of similarity in *A. hydrophila* phages 4L372D, 2L372D, 2L372X and 4L372XY were observed in the packaging, structural proteins, nucleotide metabolism, and lysis modules. The *A. rivipollensis* phages 2D05 and 4D05 showed the most similarities except in some hypothetical protein and packaging regions. Phages 44572 and 4L372X did not exhibit obvious similarities with the other phages.

The genome comparisons toward the public database showed that there were two *Aeromonas* phages (*Aeromonas salmonicida* phages 56 and 51, isolated in France [18,32]) related to *Aeromonas rivipollensis* phages 2D05 and 4D05, with above 60% coverage and 88.31% identity. The major differences in predicted functional genes between 2D05/4D05 and Phage 56/51 were small/large terminase subunits, a tail fiber protein, and ATPase. The genome of the other phages isolated in the current study shared little nucleotide-level identity with bacteriophage genomes available in the GenBank database. For example, three small sections of 44572 genome (449bp, 49,913–50,827; 124bp,18,665–18,803; 137bp, 16,602–16,708) showed 76%, 90% and 83% identity with *Salmonella* phage SE131, *Aeromonas* phage AhSzw-1, and *Vibrio* vulnificus strain FORC_036 plasmid. These regions contained the sequence of capsid and scaffold protein genes, hypothetical protein genes with unknown functions, and two tRNA genes, respectively. One small section of the 4L372X (357bp, 24,099–24,648) genome showed 79% identity with *Aeromonas* phage vB_AsaM-56, which contains a fragment of recombinational DNA repair protein gene [32].

### 3.5. Core Gene Analysis

**Figure 3:**
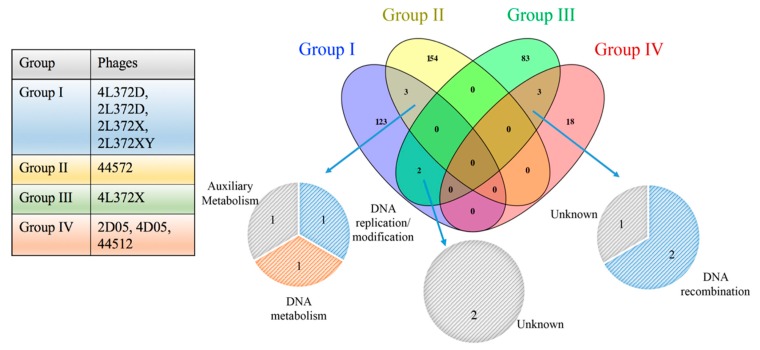
Shared core-clusters between or within different groups. The colors in the table and venn diagram indicates different groups. The colors in small pie charts represents different functions of core-clusters.

The pan-genome of nine phages (including all the ORFs from nine phage genomes) was grouped into 681 clusters. No cluster contained ORFs from all nine phage genomes. For further analysis, we grouped the nine phages according to their genome homology. The grouping information and core clusters within and between each group are shown in Figure 3.

There was no core-gene detected in the six *A. hydrophila* phages (intersection of group I, II and III) using gethomologues [28], while 18 core-genes were identified in *A. rivipollensis* phages (group IV). When we looked into the phages in different genome sizes, three core-genes were identified in larger genomes (intersection of group I and II), and another three core-genes were identified in small genomes (intersection of group III and IV) as well. The core-genes conserved in larger genomes were genes encoding a PhoH-like protein, primase/helicase, exonuclease, while the smaller genomes contained a NinG recombination protein, a NinB recombination protein, and a hypothetical protein with an unknown function.

The gene annotation functions of the nine phages are listed in Table S1. Phages 4L372D, 2L372D, 2L372X, 4L372XY and 44572 were likely virulent phages, since none of these phages contained any protein-encoding genes with significant homology to integrases, or recombinases. Gene annotation of these phages did not identify any known virulence genes or antibiotic resistance genes. Therefore, these four phages have the potential to be candidates for further phage therapy research.

### 3.6. ANI and Conserved Protein Analysis

**Figure 4:**
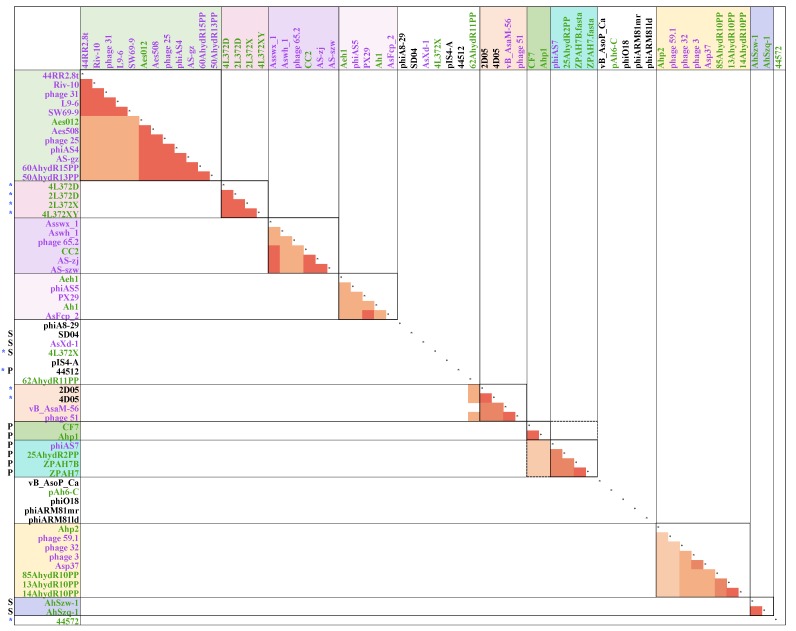
Sketch map ofaverage nucleotide identity (ANI) and conserved protein analyses of the 60 *Aeromonas* phages. The results above cutoff (ANI >50% and conserved protein >40%) are shown in orange to red. Label colors indicated different host species (purple for *Aeromonas salmonicida* subsp. *Salmonicida*; green for *Aeromonas hydrophila*; black for other *Aeromonas* species). The phages isolated in this study are indicated with a blue star, and phage morphology is indicated by the character “P” or “S”, representing *Podoviridae* or *Siphoviridae*, respectively; phages without “P” or “S” were *Myoviridae*. “*” show nine phages isolated in this study. The resulting ANI and conserved protein numbers were omitted from this table and can be found in the Supplementary Materials Table S2.

To examine how the newly isolated phages are related to previously described *Aeromonas* phages, the average nucleotide identity (ANI) and conserved gene product analyses were performed (Figure 4). Fifty-one complete genomes of *Aeromonas* phage available in GenBank and nine newly isolated phage genomes were included. Based on the result, *A. hydrophila* phages 4L372D, 2L372D, 2L372X, 4L372XY were grouped in the same cluster. *A. rivipollensis* phages 2D05 and 4D05 were grouped with *A. salmonicida* subsp. *salmonicida* phages 51 and 56, whereas *A.hydrophila* phages 4L372X, 44572 and 44512 were split with the other phages and formed singletons. Phages that infect the same host species were not clustered together in some of the clusters; for example, cluster 44RR2.8t, cluster CC2, cluster Aeh1, cluster Ahp2, cluster phiAS7, *A.hydrophila* and *A.salmonicida* phages were clustered together closely. From a morphology perspective, six *Podoviruses* (Ahp1, CF7, 25AhydR2PP, phiAS7, ZPAH7B and ZPAH7) were clustered together. The five *Siphovirus* (AhSzw-1 and AhSzq-1, SD04, AsXd-1, 4L372X) formed one cluster and three singletons.

In previous studies, 50% ANI and 40% conserved proteins cutoff together with >50% genome synteny have been used to determine phage groups [33,34]. The minimum ANI between phages within Clade 44RR2.8t was 71.35%, and 94.18–97.9% within each sub-cluster. The minimum ANI between phages within Clade CC2 and Aeh1 were 70.72% and 74.06%, respectively. The conserved protein analysis showed a similar trend with ANI results. Noticeably, the ANI and conserved proteins were above cutoff with about 65% ANI and 49.02–68.97% conserved protein. However, the genome synteny between CF7/Ahp1 and the phiAS7 cluster was below 50%.

**Figure 5:**
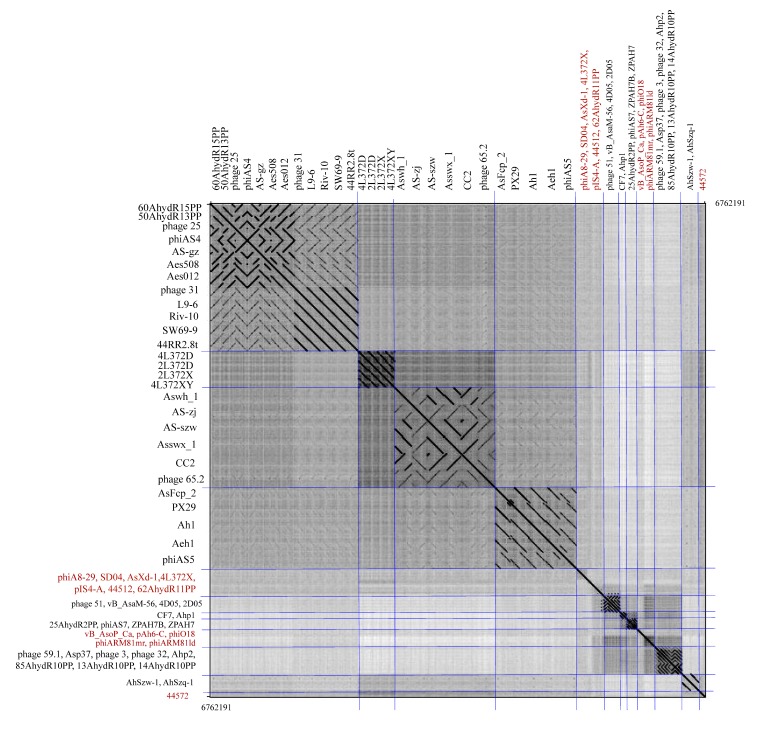
Dot plot analysis of 60 *Aeromonas* phage genomes. Blue lines separate phage clusters. A dot plot was produced using Gepard at a word size setting of 10. The 13 singletons were labeled in red color.

A dot plot analysis of 60 *Aeromonas* phage genomes was performed using Gegard [29]. The results strongly support ANI and conserved protein analyses and show similar diversity; 60 *Aeromonas* phages formed nine clusters and thirteen singletons (labeled in red) (Figure 5).

### 3.7. Phylogenic Analysis of terL

**Figure 6:**
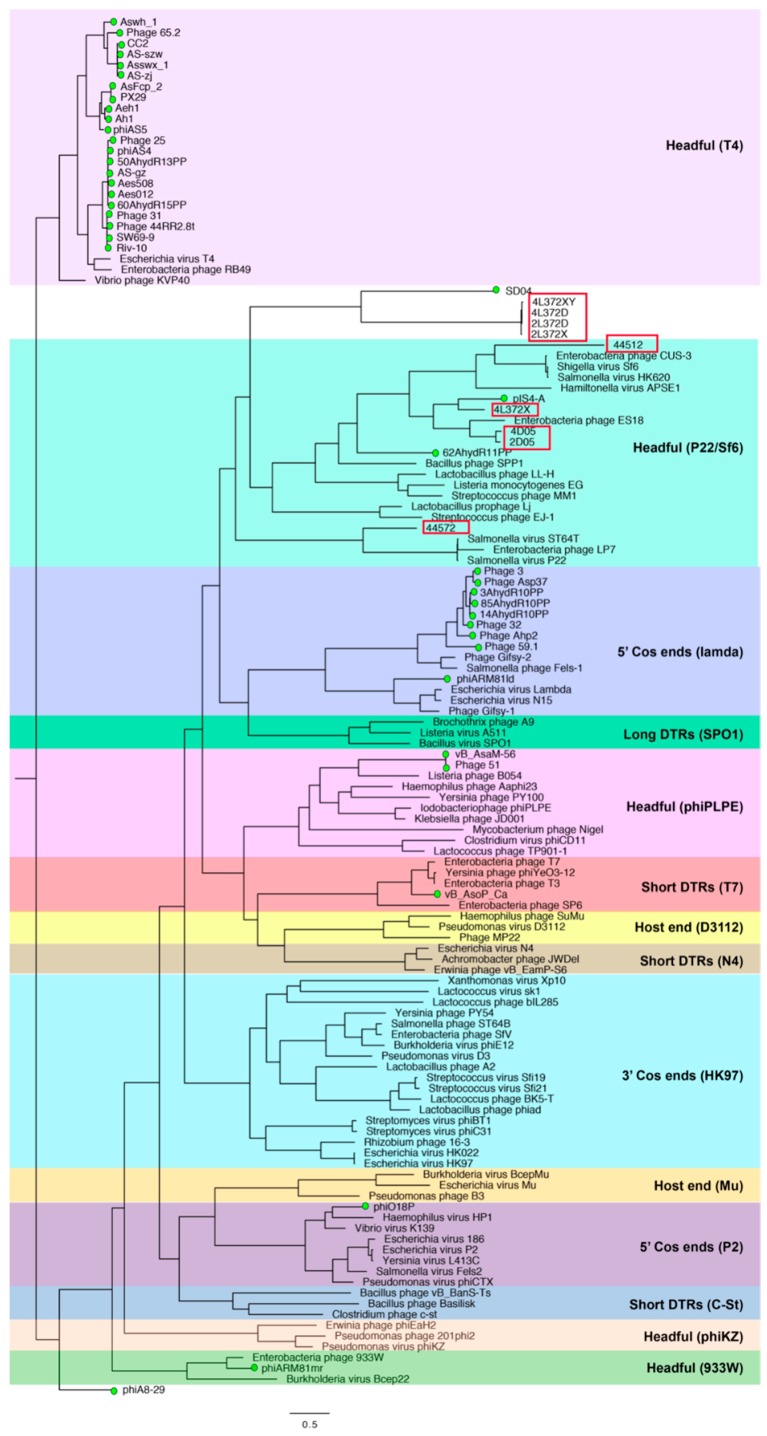
Phylogenetic tree of large terminase subunit (*terL*) amino acid sequences. The sequences of 80 reference phages with the experimentally identified packaging strategy were selected based on previously published studies. Forty sequences of *Aeromonas* phages with annotation of *terL* were included. All sequences were aligned by PROMALS3D [26]. The tree was drawn based on the Maximum-likelihood algorithm using model VT+F+R6 (selected by IQ-TREE) and 1000 ultrafast bootstrap using the IQ-TREE web service. The tree was midpoint rooted by FigTree version 1.4.3 (http://tree.bio.ed.ac.uk/software/figtree/). The phages isolated in this study are in red frame, and other *Aeromonas* phages annotated with *terL* are marked with a green circle dot.

To analyze the packaging mechanism of the nine phages isolated in this study, the method of a previous study was followed [18,25]. A phylogenetic tree was generated using *terL* protein sequences of 80 reference phages with experimentally identified packaging strategies together with *Aeromonas* phages with an annotated *terL* gene (Figure 6). The resulting tree was consistent with previous studies [18,25]. Five phages isolated in this study (phages 2D05, 4D05, 4L372X, 44512, 44572) were clustered with P22/Sf6 headful phages, which indicates that the five phages possibly adopted a similar headful packaging strategy to P22/Sf6 phages. The other four phages (phages 4L372XY, 4L372D, 2L372D, 2L372X) did not cluster with any phages with a predicted or experimentally confirmed packaging strategy. 

### 3.8. Protein Cluster Analysis of 60 Aeromonas Phages

**Figure 7:**
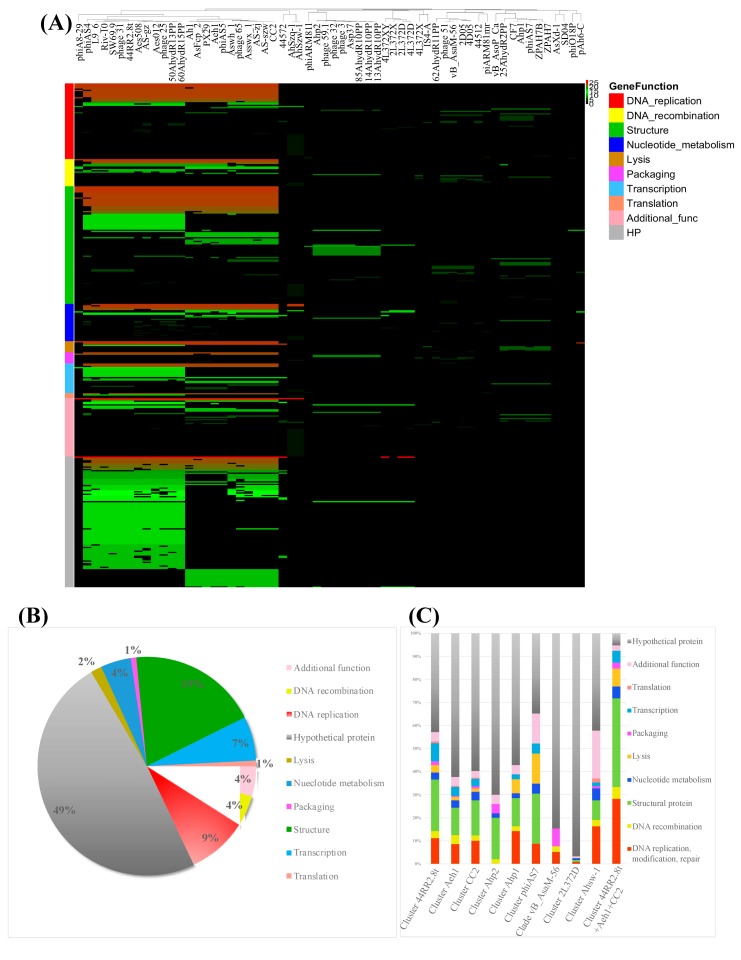
Protein cluster analysis of 60 *Aeromonas* phages. (**A**) Truncated and zoomed picture of the heatmap of 60 *Aeromonas* phages pan-clusters. (original picture is provided in supplementary document Figure S1). (**B**) Functional distribution of core clusters conserved among more than ten phages. (**C**) Functional distribution of core proteins conserved in each phage cluster. The color band corresponds to different functional categories.

To further understand the taxonomy and evolutionary process of *Aeromonas* phages, a protein cluster analysis of *Aeromonas* phages was performed. The pan-genome of 60 *Aeromonas* phages were grouped into 3091 protein clusters, including 1572 singletons. The protein clusters were conserved in multiple phage groups and those specific to subgroups were identified. There was no protein cluster identified to be encoded by all 60 phage genomes. The most prevalent clusters were the protein sequences of DNA ligase and hypothetical proteins with unknown functions, which were identified to be conserved in 28 phage genomes (Table S6). The DNA ligase was encoded in the genome of phages corresponding to clusters 44RR2.8, CC2, Aeh1 and 4L372D in Figure 4. The gene with an unknown function was conserved among phages in clusters 44RR2.8, CC2, Aeh1 and the genome of 44572, 4L372D and 2L372D. Phages in Clusters 44RR2.8t, CC2 and Aeh1 were closely related based on protein cluster analysis (Figure 7A and Figure S1).

Generally, the protein cluster analysis result was consistent with the result of ANI and dot plot analyses. Phages located in the same cluster in Figure 4 shared more conserved protein clusters. The number of protein clusters shared among each phages cluster is shown in Table 4. The 23 phages in cluster 44RR2.8t, CC2 and Aeh1 shared 39 conserved proteins. Within each phage cluster of 44RR2.8t, CC2 and Aeh1, the phages shared 126, 210 and 211 protein clusters respectively.

The functional categories of protein clusters conserved in more than ten phages are shown in Figure 7B, and 49% of the conserved proteins had an unknown function. The function of core proteins of each phage clusteris shown in Figure 7C. The protein clusters differentiated between phage cluster 44RR2.8t and CC2 plus Aeh1 were mainly host lysis and attachment-related proteins (holin, tail fiber and baseplate proteins), transcription-related proteins, and proteins with additional functions. Among them, holin was conserved in phage cluster 44RR2.8t, not encoded in cluster CC2 or Aeh1. The tail fiber and base plate proteins were mainly clustered into different types corresponding to their phage group. This might explain the wide host range of phages in cluster 44RR2.8t, as reported in a previous study [18]. Vincent et al. [18] reported that *A. salmonicida* phages 44RR2.8t, SW69-9, phage 31, L9-6, Riv-10 (in cluster 44RR2.8t) had a “broadest host range” (>44 of 65 *A. salmonicida* host could be lysis). The different strategies of attachment and host lysis might explain the host range discrepancy between these phages. The different core proteins between phage cluster CC2 and Aeh1 were DNA polymerase, prohead core protein, head outer capsid protein, hinge connector protein, RNA polymerase, rIIa protector protein from prophage-induced early lysis, and some hypothetical proteins with unknown functions.

### 3.9. Host Range Analysis

The host range of five *A.hydrophila* phages was tested using six *A. hydrophila* strains purchased from China General Microbiological Culture Collection (CGMCC), Agricultural Culture Collection of China(ACCC) and China Center for Type Culture Collection (CCTCC). The five phages show specificity to the host used to isolate them and cannot lyses other tested *A. hydrophila* bacteria (Table 5). Phage 44572 could cross-infect *A.rivipollensis* 4512.

## 4. Discussion

Before this work, there were 51 genomes of *Aeromonas* phages in total in GenBank, 17 of which infect *A.hydrophila* phages (on 1 April 2019). In this study, we further extended the picture of *Aeromonas* phage diversity by isolating and sequencing nine novel phage genomes—which formed a new phage cluster and three singletons—from southwest China. The phage genomes were all annotated and deposited in GenBank (accession numbers are shown in Table 2).

Although the nine novel phages were isolated from the same site using four host strains belonging to two species, they appeared to be highly diversified on morphology and genome level (including genome length, GC%, genomic homology and evolutionary relationships), even for the phages infecting the same host species or strain. According to comparative genomic and core-gene results, *A.hydrophila* phages 4L372X and 44572 did not show notable similarities with other *A.hydrophila* phages isolated in this study, and therefore no core genes were identified in *A.hydrophila* phages. However, connections between *A.hydrhophila* phage 4L372X and three *A.rivipollensis* phages in genome length and conservative genes were observed, and three core-genes were detected. *A.rivipollensis* phages 2D05 and 4D05 showed high nucleotide similarities with *A. salmonicida* subsp. *Salmonicida* phages 51 and 56 (71% and 68% respectively). The four phages were clustered together in ANI, conserved protein and dot plot analysis, but split in the *terL*-based tree, which suggested that their *terL* gene had undergone horizontal gene transfer [35]. To clarify, one may notice that the *terL* gene was annotated in all nine phage genomes, whereas no core-gene was identified using gethomologues software in Figure 3. This is due to the low similarities in the terL sequences of the nine phages. In the BLASTP [21] alignment results of terL, the coverage of nine phages varied 5–100% and e-value ranged from 8× 10^−139^ to 8.3, which did not meet the software default cutoff of minimum 75% coverage and 1× 10^−5^ in BLAST pairwise alignments.

The result of the core-gene analysis of the nine phages also demonstrated the difference of functional contents conserved in small and larger genomes. Interestingly, the core-genes shared within small genome groups had mainly DNA-recombination-related functions, while in the larger genome group, the core-gene functions involved auxiliary metabolic gene and DNA replication and metabolism. It is likely that phages with larger genomes have the capacity to harbor more genes that benefit their replication directly (e.g., DNA replication and metabolism genes) or indirectly through mediation of the metabolism and growth of their hosts (e.g., auxiliary metabolic gene) [36].

*PhoH,* the auxiliary metabolic gene conserved in larger genome phages in our study, is a host-derived auxiliary metabolic gene in phage genomes, which regulates phosphate uptake and metabolism under low-phosphate conditions [37]. It has recently been identified as an effective signature gene for examining phage diversity in marine and paddy water environments, whereas freshwater distribution of *phoH* has not been studied yet [38,39]. From our results, all five phages with larger genomes contained *phoH*, which suggests that *phoH* is also distributed in freshwater. However, there were still some phages, e.g., phages with smaller genomes isolated in this study, that did not carry *phoHin* their genome, which indicates the limited specificity of this method.

We also extended our analysis to the sequenced complete genomes of *Aeromonas* phage available in GenBank to obtain an overview of *Aeromonas* phage diversity. Vincent et al. investigated the genomes of 18 phages infecting *A. salmonicida* subsp. *salmonicida,* and provided classification based on their genomic composition but with different analytical methods [18]. In our study, we included all *Aeromonas* phages with available genome data in GenBank, which was comprised of phages infecting *A.salmonicida*, *A.hydrophila*, *A.rivipollensis* and other *Aeromonas* species. The results showed that the classification of *A.salmonicida* phages was consistent with Vincent’s result and brought additional members into classified groups. According to the recommendations of the International Committee of Taxonomy of Virus [40], the results of genome synteny, proteome and ANI analyses and the clustering of *Aeromonas* phages were consistent among the results. Therefore, we suggest widening phage 65 similar phages and Aeh1 similar phages in the *Tevenvirinae* subfamily, and propose these two groups as new genera: “*phage65virus*”, containing phage 65, CC2, AS-zj, Asswx_1, Aswh_1, and “*Aeh1virus*”, containing Aeh1, phiAS5, PX29, Ah1, AsFcp_2. We also suggest wideningthe *Secunda5virus* genera to include new members Riv-10, SW69-9, AS-gz, 85AhydR10PP, 13AhydR10PP, 14AhydR10PP, and moving 44RR2.8t from the *Biquartavirus* to the *Secunda5virus* genera [33,34,41]. Moreover, according to the high nucleotide sequence similarity and conserved protein clusters shared, we propose two more genera:“*phage56virus*”, containing phage 51, vB_AsaM-56, 2D05 and 4D05, and “*2L372dvirus*”, containing 2L372D, 2L372X, 4L372D and 4L372XY under the *Myoviridae* family. The current taxonomic proposals were only based on the above analysis on 60 *Aeromonas* phages, and phages that infect the hosts of other genera were not included in the study. As more phages are isolated, it is possible that non-*Aeromonas* phages will be added in the taxa.

A previous study [18] reported that *A. salmonicida* phages 44RR2.8t, SW69-9, phage 31, L9-6, Riv-10 showed a broad host range compared to other *A. salmonicida* phages. In the current study, the protein cluster analysis identified holin proteins, some tail fiber, and base plate proteins specific to phage cluster 44RR2.8t. Holin proteins are reported to trigger and control the degradation of the host cell wall by forming pores in the host cell membrane and allowing lysins to reach and degrade cell walls [42,43]. Tail fiber proteins are also known to relate to phage-host attachment and host range determination [44,45,46,47]. Therefore, these unique proteins encoded in the genomes of phages in cluster 44RR2.8t might be the determining factors intheir broad host range feature. Since the homologies of the above proteins were also detected in other *A. salmonicida* phages of cluster 44RR2.8t (e.g., phiAS4, AS-gz, Aes508, phage 25, 60AhydR15PP and 50AhydR13PP), these phages might potentially obtain a broad *A. salmonicida* host range as well, but further experiments need to be conducted.

The common practice of phage discovery in phage-therapy has been screening phages using the pathogenic host bacteria. The isolated phages are usually tested in the host range of the same host species, although there are reports of phages which can infect multiple host species or genera. From this study, we noticed that many clusters containing phages were reported to infect different *Aeromonas* species. This provided a hint that these closely related phages might be able to infect different host species, which could potentially benefit the use of isolated phages in the treatment of a different host. However, more experiments need to be performed to verify this.

To conclude, in this study, we conducted a genome-level analysis of nine new phages together with 51 sequenced *Aeromonas* genomes from GenBank. From the results, the phages that infect the bacterium of the *Aeromonas* genus are remarkably diversified. The newly isolated phages showed heterogeneous genomic and evolutionary relationships, although they were isolated from the same environmental sample and some of them were isolated using the same host species or strain. The conserved genes in small and larger genomes of these phages were distinct, and the phages with a larger genome tended to harbor auxiliary metabolic genes. The 60 *Aeromonas* phages formed nine clusters and 13 singletons, which was consistent using different analytical methods. According to the clustering results, we proposed a new classification scheme for the *Aeromonas* phage.

## Figures and Tables

**Table 1:** List of *Aeromonas* phages available in GenBank on 1 April 2019.

	*A. hydrophila* Host	*A. salmonicida* Host	Other *Aeromonas* Species Host
*Myoviridae*	pAh6-C, 60AhydR15PP, 14AhydR10PP, 85AhydR10PP, 50AhydR13PP, 13AhydR10PP, Aes012 (*Secunda5virus*), CC2 (T4-like), Ah1 (*Tevenvirinae*), Aeh1 (*Tevenvirinae*)	vB_AsaM-56, phage 51, phage 59.1, phage 3, Asp37, phage 32, AS-gz, SW69-9, Riv-10, PX29, phiAS5, AS-szw, phage 65.2, 44RR2.8t (*Biquartavirus*), Aes508 (*secunda5virus*), phage 25 (*secunda5virus*), phiAS4 (*secunda5virus*), phage 31 (*secunda5virus*), L9-6 (*secunda5virus*), AS-zj (*Tevenvirinae*)	phiARM81ld, phiARM81mr, phiO18P
*Podoviridae*	ZPAH7B, ZPAH7, CF7 (*Autographivirinae*), 25AhydR2PPb (*Autographivirinae*), Ahp1	AS7 (*Autographivirinae*)	vB_AsoP_Ca
*Siphoviridae*	AhSzq-1 (*T5virus*), AhSzw-1 (*T5virus*)	AsXd-1 (*Hk97virus*)	SD04, pIS4-A (*Pis4avirus*)
Other family or unclassified	Ahp2 (ssDNA viruses/*Inoviridae*), 62AhydR11PP (unclassified)	Aswh_1 (unclassified), AsFcp_2 (unclassified), Asswx_1 (unclassified)	phiA8-29 (unclassified)

**Table 2:** Characteristics of nine *Aeromonas* phages isolated in this study.

Name	Taxonomy	Host Strain	Head Diameter/Tail Length (nm)	Genome Size (bp)	ORFs	tRNAs	GC%	Plaque Diameter (cm)/Turbidity	GenBank Accession No.	Raw Reads ^b^	Clean Reads ^b^	Number of Contigs
2L372X	Myoviridae	*A. hydrophila* subsp. *hydrophila* L372	78/91	115,893	211	0	35.6	0.35 ± 0.04/clear	MK813938	4,629,843 × 2	4,260,615 × 2	1
2L372D	Myoviridae	*A. hydrophila* subsp. *hydrophila* L372	81/103	122,963	217	0	35.3	1.27 ± 0.15/clear	MK804893	4,861,451 × 2	4,577,807 × 2	1
4L372D	Myoviridae	*A. hydrophila* subsp. *hydrophila* L372	74/78	138,690	252	1	35.1	0.73 ± 0.04/clear	MK813939	150,292	26,046	1
4L372XY	Myoviridae	*A. hydrophila* subsp. *hydrophila* L372	65/90	124,279	221	0	36.2	0.32 ± 0.01/clear	MK813941	3,200,688 × 2	2,977,147 × 2	1
4L372X	Siphoviridae	*A. hydrophila* subsp. *hydrophila* L372	54/102	45,698	83	3	48.7	0.33 ± 0.07/clear	MK813940	4,616,096 × 2	4,472,870 × 2	1
44572	unclassified	*A. hydrophila* subsp. *hydrophila* 4572	165 × 51/NA ^a^	102,915	154	20	42.5	0.73 ± 0.10/clear	MK813943	5,022,347 × 2	4,682,235 × 2	1
2D05	Myoviridae	*A. rivipollensis* D05	43/65	43,233	83	0	56.4	0.87 ± 0.21/clear	MK804891	150,292^b^	10,322 ^b^	1
4D05	Myoviridae	*A. rivipollensis* D05	60/114	42,249	74	1	56.0	0.49 ± 0.03/clear	MK804892	5,849,472 × 2	5,420,148 × 2	1
44512	unclassified	*A.rivipollensis* 4512	54/NA ^a^	41,963	63	0	52.3	1.61 ± 0.14/clear	MK813942	4,420,742 × 2	4,147,413 × 2	1

^a^ NA: Not Available. The tail of phage 44572 was not detected by TEM. ^b^ The genomes of 2D05 and 4L372D were sequenced using PacBio RS II platform. The genome of 2L372X, 2L372D, 4L372XY, 4L372X, 44572, 4D05 and 44512 were sequenced using Illumina HiSeq 2000 and paired-end reads were generated. Detailed sequencing and assembly statistics were in Supplementary Materials Table S5.

**Table 3:** Nucleotide identities of nine phages using BLASTn pairwise alignment [22]. The sequence coverages and identities (in brackets) are listed.

	4L372D	2L372D	2L372X	4L372XY	44572	4L372X	44512	2D05	4D05
4L372D	*								
2L372D	86% (99.81%)	*							
2L372X	68% (99.92%)	82% (99.81%)	*						
4L372XY	64% (99.12%)	62% (98.51%)	72% (98.5%)	*					
44572	-	-	-	-	*				
4L372X	-	-	-	-	-	*			
44512	-	-	-	-	-	-	*		
2D05	-	-	-	-	-	-	19% (93.96%)	*	
4D05	-	-	-	-	-	-	18% (98.82%)	86% (98.59%)	*

‘-’ indicates no significant similarity. ‘*’ indicates 100% coverage and identities.

**Table 4:** Number of conserved proteins of each phage cluster.

ClusterName	ClusterMembers	No. of Shared Proteins	Percentage of Shared Proteins on Average Number of Proteins on Phage Genomes
44RR2.8t	44RR2.8t, Riv-10, phage 31, L9-6, SW69-9, Aes012, Aes508, phage 25, phiAS4, AS-gz, 60AhydR15PP, 50AhydR13P	126	50.96%
Aeh1	Aeh1, phiAS5, PX29, Ah1, AsFcp_2	210	61.73%
CC2	Asswx_1, Aswh_1, phage 65, CC2, AS-zj, AS-szw	211	51.13%
Ahp1	Ahp1, CF7,	49	100%
phiAS7	phiAS7, 25AhydR2PP, ZPAH7B, ZPAH7	23	57.5%
Ahp2	Ahp2, phage 59.1, phage 32, phage 3, Asp37, 85AhydR10PP, 13AhydR10PP, 14AhydR10PP	50	60.24%
vB_AsaM-56	2D05, 4D05, vB_AsaM-56, phage 51	39	48.15%
2L372	4L372D, 2L372D, 2L372X, 4L372XY	123	54.61%
Ahsw-1	AhSzw-1, AhSzq-1	116	81.69%

**Table 5:** Host range analysis of five *A. hydrophila* phages.

	CGMCC 1.1801	CGMCC 1.1816	CGMCC 1.2017	ACCC 01748	ACCC 10482	CCTCC AB209165	L372	4572	4512	D05
2L372D	−	−	−	−	−	−	+	−	−	−
2L372X	−	−	−	−	−	−	+	−	−	−
4L372X	−	−	−	−	−	−	+	−	−	−
4L372XY	−	−	−	−	−	−	+	−	−	−
44572	−	−	−	−	−	−	−	+	+	−

‘−‘ represent negative results; ‘+’ represent positive results.

## Supplementary Materials

The following are available online at https://www.mdpi.com/1999-4915/11/7/615/s1, Figure S1: Original picture of the heatmap of 60 *Aeromonas* phages pan-clusters. Table S1: Gene function table showing the location of genes with identified functions in the genomes of the nine isolated phages. Table S2: ANI and conserved protein results of 60 *Aeromonas* phages. Table S3: GenBank accession numbers of *Aeromonas* phages used in this manuscript. Table S4: GenBank accession numbers of terL protein used in this manuscript. Table S5: Sequencing and assembly statistics. Table S6: Pan-clusters list of 60 *Aeromonas* phages.
