# Supplementary material for: Nine Novel Phages from a Plateau Lake in Southwest China: Insights into Aeromonas Phage Diversity
Source: Viruses. 2019 Jul 5;11(7):615. doi: 10.3390/v11070615 (PMC6669705; doi:10.3390/v11070615)
Supplement: Supplementary file 1 [file viruses-11-00615-s001.zip › Supplementary/Table S1.docx]

**Table S1. Gene function table showing the location of genes with identified functions in the genomes of the nine isolated phages.**
