# Supplementary figures and images for: Nine Novel Phages from a Plateau Lake in Southwest China: Insights into Aeromonas Phage Diversity
Source: Viruses. 2019 Jul 5;11(7):615. doi: 10.3390/v11070615 (PMC6669705; doi:10.3390/v11070615)

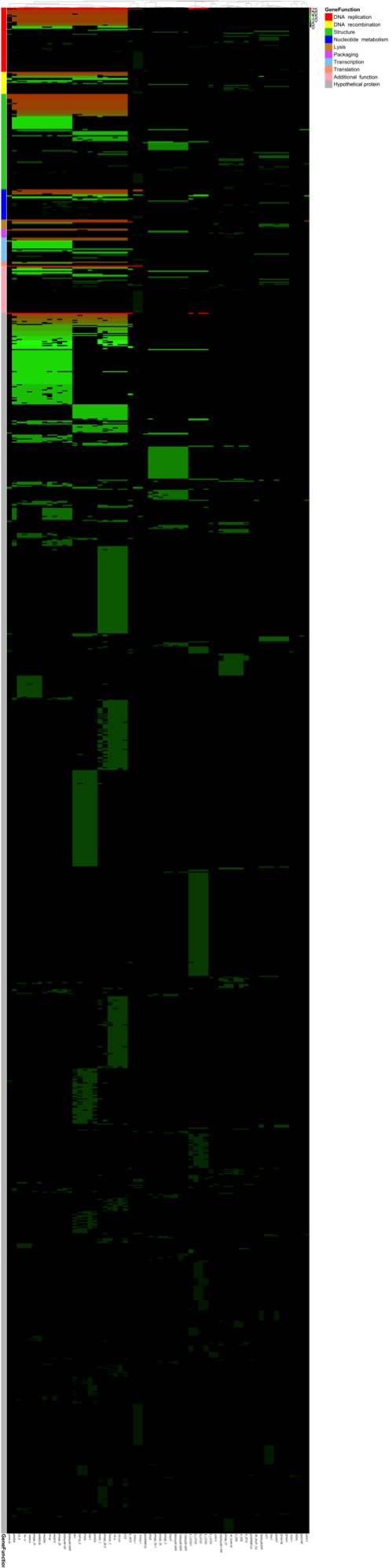


Figure S1. Original picture of the heatmap of 60 *Aeromonas* phages pan-clusters.

Supplement: Supplementary file 1 [file viruses-11-00615-s001.zip › Supplementary/Figure S1.docx]
